# A System of Practical Medicine, Comprised in a Series of Original Dissertations

**Published:** 1840

**Authors:** 


A System of Practical Medicine, comprised in a Series of Original Dissertations. Arranged and Edited by *Alexander Tweedie*, M.D. F.R.S. Octavo, pp. 440. Whittaker, April, 1840.

We have some doubt as to the propriety of the term "*original* dissertations" in the title-page of this work. If dissertations on fever, inflammation, and many other diseases be *original*, they must necessarily omit nine-tenths of the accumulated experience of the medical world, from the days of Hippocrates to the present time. But the fact is that the work under review is a compilation, and a compilation like that of Copland and its predecessor the Cyclopædia, interlarded with the peculiar views of the individual compiler. This observation implies no censure or discredit—quite the contrary. In works of this kind every thing depends on the judgment and industry of the compiler. Neither judgment nor industry will do alone. They must be combined. They can hardly ever be expected, however, in due proportion in the same individual. A high rate of judgment based on experience, often leads to laziness of research—while incessant researches among books often bewilders even the best judgment.

If the succeeding volumes of the "Library of Medicine" keep pace in merit with this first one, we predict that the whole will attain a high reputation and an extensive circulation. We are free to confess, indeed, that in these dissertations, or whatever else they may be called, the materials selected from preceding writers are so well worked up, so amalgamated, and so artfully moulded, as to resemble very much *Original Essays*. The great majority of the writers in this volume stand high in the opinion of their brethren, and though several of them are actively engaged in the exercise of an extensive and fatiguing practice, they do not appear to have spared the midnight oil or the labour of cogitation, in the articles allotted to them. It cannot be expected that we can analyze an elementary work, however artfully cast in the moulds of originality; yet there are more than one or two dissertations in this volume, to which we shall dedicate separate articles, as soon as we can spare space and time.

The "Pathological Introduction" by Dr. Symonds, comprises in the space of 52 pages, a body of valuable facts and observations which, by a practised writer, might have been spread over three goodly octavos, if well swelled out with long quotations. The object of it is to facilitate the study of diseases by exhibiting a brief view of their more simple or elementary forms. There is no doubt that the student is greatly embarrassed on the very threshold of his studies, by numerous terms which require, each, a dissertation rather than a dictionary for explanation. Thus, what idea can be formed of "congestion," "inflammation," "hypertrophy," "plethora," &c. without a full explanation of the nature and causes of these states or conditions of the system. One or two extracts will serve as specimens of the manner in which our author has executed this part of the work.

"*Revulsion* is exemplified in such cases as the following:—congestion of the cerebral capillaries may be removed by congestion in the hæmorrhoidal vessels; subacute inflammation of the bronchial membrane by a cutaneous eruption; dropsy of the peritoneum by diarrhœa; a spasm of the bronchial fibres by dysphoria, or fidgets. We have known an inveterate asthma which had existed for years superseded by tic-douloureux. Blisters, issues, and setons, are artificial measures intended to imitate the curative operation of one diseased action upon another. Hence they are called revulsives, or counter-irritants. The establishment of a secondary affection to the exclusion of the primary is manifestly the very opposite of sympathy, in which there is a reciprocal maintenance of the diseased conditions. It occurs more frequently in parts of similar anatomical constitution, as when irritation of the skin relieves that of a mucous membrane. We think, also, that the relation is oftener manifested between actions similar in kind: thus a rubefacient is better adapted for the removal of internal congestion than of a suppurative disorder, for which a seton or issue would be more appropriate." 6.

"*Metastasis* is often confounded with revulsion, but it differs from the latter in the fact, that the second action is not observed until the first has disappeared. The name implies change of place; and, at the time of its introduction, the prevalent belief was, that when a disorder changed its seat, a morbific matter had been transplanted from one part to another. The subsidence of a cutaneous eruption may be followed by inflammation of the bronchial membrane, and a similar relation may be observed between the latter and inflammation of the mucous surface of the intestine. The sudden cessation of hæmorrhois may induce disorder of the brain, such as a transient giddiness, or a fatal apoplexy. Metastasis is observed to occur more frequently in some diseases than in others, as in the instances of gout and rheumatism flying from part to part.

The pathology of these affections is somewhat too complicated to be entered upon at present. We must content ourselves with stating what we consider to be the law of metastasis; namely, that the liability of a disease to metastasis is in an inverse ratio with its dependence upon local causes; whence it may be inferred, that the occurrence implies a fault in the general system. In a plethoric subject, the removal of a determination of blood to a particular part by means which do not reduce the quantity of nutritive fluid, is very easily succeeded by similar disease in some other organ. Congestions dependent upon irritability of the nervous system are likewise migratory. We shall find the principle of very extensive application in pathological inquiries. There are cases, however, in which the cessation of a purely local affection is almost certainly followed by disease elsewhere. We allude to those in which a disease has been so long fixed in an organ as to have become a sort of necessary function to the whole system, or in which other parts have gradually become accommodated to it, so that upon its removal the harmony is again disturbed. Thus an ulcer may have existed so long that either the whole quantity of the blood, or the relative distribution of it to the several organs, has been adjusted to the action on the morbid secreting surface. The healing of the ulcer is, therefore, very likely to cause excessive determination to some internal organ; and that such events often take place in this order is matter of every day's experience." 7.

The author then proceeds to consider "diseases of the capillary system," under the heads of "disordered circulation"—"diseased secretion"—"diseased nutrition"—"diseases of the blood"—ending with "diseases of nerves and contractile fibres"—all of which important subjects, he handles in a very masterly manner. The language is so terse, and the reasoning so close, that the condensing cylinder of the most practised analytical reviewer would be utterly foiled in any attempt to compress the matter into smaller space. One more specimen is all that we can offer from this very clever introduction. Although the subject of "inflammation" occupies 61 pages of the work itself,—and that from the pen of Dr. Alison, yet the following graphic sketch of this foundation of almost all diseases, comprises nearly the whole of what we know respecting its elementary nature.

"Inflammation consists not only in engorgement of the capillary vessels of the part, but also in the arrest and coagulation of the circulating fluid. It occurs under circumstances precisely similar to those which favour or produce congestion; and, in fact, the latter is the incipient stage of inflammation, though it may also exist (as we have seen) as a separate lesion. Inflammation moreover may be divided, like congestion, into active and passive, according to the mode of its production, and to the symptoms which accompany it. Congestion is apt to be converted into inflammation, when its causes act with great intensity, that is, 1. when the blood is attracted in so overpowering a quantity that the ordinary powers of the capillary circulation are unequal to the disposal of it; 2. when the vitality is so low that the motion is not only retarded, but suspended; and, 3. when the venous obstruction is so considerable as to have the same effect. Inflammation is also more likely to occur when the accumulation has not been relieved by the exudation either of blood or of serum, nor by an increase of the normal secretion of the part.

The process of inflammation has been carefully observed by the aid of the microscope; and these are the principal phenomena:—At first, the globules of blood are seen upon the application of the stimulus to move more rapidly, and the currents are somewhat smaller in diameter; but this is soon succeeded by dilatation, and near the point of irritation the motion is slower. In the surrounding parts blood begins to accumulate, and there is a general afflux from the more distant points of the reddened surface to the point of irritation as a centre. At this time the particles may be seen to flow in a direction retrograde to the usual course, and to find channels not previously observed. The next and most important phenomena are the stagnation of the blood in the central part, its coagulation, and the disappearance of the globules in a confused mass. Inflammation is now established; the colour of the part varies, according to the stage of the process, from vivid red to dull crimson or black. The arteries leading to the spot are more distended than usual; and they have been proved by Dr. Alison to lose much of their tonic contractility. The veins leading from the inflamed member yield a larger proportion of blood than those of sound parts.

It has been long a warmly disputed question, whether inflammation is to be regarded as increased action. The facts just detailed are obviously quite incompatible with the notion of increased action (*i. e.* contraction) of vessels. But if increased action refers to the quantity of blood in the tissue, and to the state of the neighbouring parts, it certainly must be predicated of inflammation. The great afflux of blood, the heightened colour, the augmented bulk, the exalted sensibility, the increase of temperature, the throbbing arteries and distended veins, not to mention the subsequent products of serum, fibrin, and pus, present a collection of phenomena which it is difficult to separate from the idea of increased action or commotion; but it is quite certain that there is weakened action of the vessels, with diminished function of the tissue. As inflammation will be treated of more at large in another portion of this work, we have only attempted to say as much as seemed requisite for pointing out the relations of this with other pathological conditions." 18.

The subject of "INFLAMMATION" in all its details, has been assigned to Dr. Alison, and we need hardly say that few men could treat the subject with greater ability. Having exhibited an extract containing an epitome of the theory of inflammation, we are here tempted to introduce another, and a longer one, discusssing the pros and cons respecting our great remedy for the disease—namely bloodletting. The "cautions" here detailed, are well worthy of attention from old and young practitioners.

"But the principal cautions which it is necessary to keep in mind, as to the use of bloodletting in inflammatory diseases, have reference, not simply to the subsequent effects of the evacuation on the system, but to the alteration to be expected from it on the progress of the existing disease; and in this view we must always carefully attend—1. to the *period of the disease* at which we are to use the remedy; 2. to the kind of the inflammation; and 3. to the complication which may exist of inflammation with other diseases.

1. When we say that the period of the disease, even in cases of healthy inflammation often decidedly contra-indicates, and still oftener makes us doubtful as to the result of bloodletting, we do not mean merely the number of days from the first decided attack of the disease (although that always demands attention;) but we must attend particularly to the proofs of effusion or disorganisation consequent on the inflammation, having already made such progress as to indicate that the alteration of structure already effected, rather than the alteration of action which leads to it, demands our chief attention. And in general, as already remarked, this may be apprehended when we see a *manifest change in the constitutional or febrile symptoms,* attended with *continuance or increase of the local symptoms.* When the pulse has become slow or irregular, at the same time that the pain of head has passed into delirium or coma, in phrenitis or hydrocephalus; when it has become soft and compressible, or very frequent, or when the fever has taken the form of hectic, in inflammations within the chest, while the cough, or the dyspnœa has continued and increased; when rigors have supervened on hepatitis; when a soft and compressible, or very frequent pulse, and cold sweats have taken place in enteritis, without relief of the bowels or abatement of the tenderness of the abdomen, thoracic respiration, and vomiting; we may always suppose that effusions have taken place to a considerable extent, and that if the inflammation exciting them has not subsided, at least the febrile reaction by which that inflammation had been supported, and on which bloodletting could exert its chief powers, has so far abated, that the time for active depletion is nearly over. If recovery is possible after this period, a long and slow process must be gone through before it can be perfected; and this will require a certain strength of vital action, and may be frustrated by any means which further depress the vital powers; nay it may, in many instances, be obviously promoted by means which excite the system generally, and stimulate and strengthen the circulation.

In many such cases, more definite information is attainable, particularly in the case of inflammation within the chest, whether affecting the bronchiæ, the substance of the lungs, the pleura, the pericardium, or inner membrane of the heart,—the indication given by examination of the chest and of the sputa, and by auscultation and percussion, prove the extent of effusion, and the degree in which the play of the lungs or heart is impeded by it; and these, taken along with the state of the pulse, heat of skin, and general strength, may often enable us to speak with much confidence as to the question,—always presenting itself in the advanced stage of these diseases,—whether there is more danger from weakening the circulation by bloodletting, when such impediments to the action of parts within the chest already exist, and can only be remedied by a slow natural process of absorption, or from allowing such inflammation as still exists to go on, unchecked by farther loss of blood.

2. That inflammation may exist, of a nature not to be subdued, even to be aggravated, by bloodletting or other evacuations, is quite certain from such experiments as those of Magendie as to the eye, and of Gendrin as to the stomach; in which the kind of inflammation of mucous membrane formerly mentioned was brought on by inanition, and could only be relieved by fuller nourishment, restoring the strength of the circulation, and probably restoring to the mucous membrane its natural protecting mucus ; and that the kind of inflammation which is recognised in a patient affords very often a reasonable ground of objection to full bloodletting, is sufficiently obvious when we attend to the known history of scrofulous, rheumatic, and gouty inflammation. In the first of these, it is true, that on occasion of a recent inflammatory attack, when the symptoms approach most nearly to those of healthy inflammation, we have every reason to believe that bloodletting is often of the most essential importance, preventing aggravation of disease already existing, or arresting disease which would otherwise be established. But it is equally true that scrofulous inflammation is less under the influence of bloodletting than healthy inflammation ; and farther, that scrofulous diseases occur chiefly in weakly persons, in those whose mode of life in early youth has been debilitating, and in those recently weakened by any considerable evacuations. Therefore, by full and repeated bloodletting in scrofulous cases, while we make little impression on the inflammation that exists, we incur a great risk of so far lowering the constitution as to make it more liable than previously to fresh attacks of inflammation, or to other scrofulous diseases, perhaps not inflammatory in their origin.

Again, the recorded experience of all ages informs us (whatever we may conjecture as to the explanation of the fact) that the inflammation both of rheumatism and of gout is very liable to metastasis, and that, although it may often be moderated (particularly that of acute rheumatism in a healthy constitution) with very good effect by evacuations, yet it is by no means desirable that it should suddenly recede from the extremities ; because if it does, inflammation in a more vital organ, or in the case of gout, a kind of internal neuralgia, even more immediately dangerous, is very likely to follow.

In the case of erysipelas, to a certain degree, and in that of all the specific inflammations of the skin already noticed, in a much greater degree, the nature of the inflammation may also be urged as a reason against full bloodletting, and in favour of the strictly 'expectant practice ;' but these are cases either of inflammation without fever, or of inflammation complicated with another and generally more formidable disease, falling therefore under the next head.

3. The complications of inflammation which often contra-indicate bloodletting, and always impose the necessity of caution in regard to it, are in a general view of two kinds ; that with other febrile and particularly contagious diseases, and that with chronic and particularly organic diseases.

In regard to the complication of inflammation with idiopathic fever or with the contagious exanthemata (in which we include erysipelas), the general principle is, that such inflammation, whether of the kind that is essential to and characteristic of the disease, or of that which is only an accidental concomitant, is never the sole, and seldom the chief, cause of danger. The body is under the influence of a poison, generally absorbed from without, which gives a peculiar character to the inflammation, and likewise excites a peculiar form of fever, often very dangerous when the inflammation, external or internal, is trifling. In the course of the disease, the poison, after being enormously multiplied, by some mysterious process, is expelled from the body. Whether the inflammation is part of the process by which this expulsion is effected, is indeed doubtful, but it is certain, that in most of these diseases, the inflammation, at least that which is characteristic and peculiar to the disease, cannot be prevented from running a certain course without imminent danger to life.

The danger in the course of these diseases depends often mainly on the depressing effect of the morbific poison, gradually influencing the system at large and especially the fundamental function of circulation, and producing typhoid fever ; but it often depends on the combination of the depressing influence with inflammation, internal or external ; and sometimes it depends so much on the intensity of the inflammation and so little on any general depression of the powers of life, that the disease demands and bears evacuations nearly as in idiopathic inflammation. In judging of the degree in which the danger of individual cases depends on the one or the other cause, there is of course much room for the exercise of judgment and discretion. One general observation may be made, which is of great practical importance, that in all such complex cases, where contra-indications exist, if bloodletting is to be practised (and in the accidentally concurrent inflammations in many cases of such diseases it is highly beneficial), it should be practised as early as possible, in order that it may be as small as possible; all experience informing us that a very moderate loss of blood in the early stage of inflammation will often produce much more effect on the extension and course of the disease than a much larger quantity at an advanced period.

There is another element which must always be taken into consideration here, which is quite peculiar to such diseases, viz. the nature of the prevailing epidemic; for it is the general result of the observation of medical men in different ages, that, in different epidemics, the type of the same disease so far varies, that the local inflammations may be more frequent and dangerous in the generality of cases occurring in one, and the general typhoid state in those occurring in another. Thus it is the general result of the experience of the present writer and, he believes, of most practitioners who have seen much of the epidemic fevers prevalent in Scotland from 1816 till 1820, and again of those prevalent since 1826,—that bloodletting was both more demanded from the firmness of the pulse and the urgency of local symptoms, and better borne at the former time; and that the danger much less frequently depended on mere depression of the circulation; and again, that, in the latter epidemics, this last part of the symptoms has been much more generally urgent, the use of stimulants has often appeared much more important and beneficial; and that full bloodletting, even early in the disease, has often appeared to exert a very injurious influence over its subsequent progress. Similar observations have been made on different epidemic visitations of all the febrile and contagious diseases.

Of the caution in regard to bloodletting which is imposed by the presence of chronic, and especially of organic disease, we may merely enumerate the cases of inflammation of the lungs or bronchiæ combined with disease of the heart, or with previous long-continued asthmas and its usual attendant, emphysema of the lungs; and again, of inflammation within the abdomen, whether of the serous or mucous membrane there, combined with organic disease of the liver. Such cases are very common, and are very often farther complicated with dropsical effusion, partial or general. It is very important to be aware, and has been ascertained of late years more distinctly than formerly, that none of these complications ought to prohibit bloodletting when the inflammatory symptoms are recent, and the circulation tolerably firm and vigorous. But it is obvious that, in such cases, the system is permanently under the influence of a cause which prevents it from recovering its natural strength after any great evacuation, as it otherwise would do. And in several such cases, a more special cause of danger from much loss of blood may be pointed out, particularly in the cases of advanced bronchitis and emphysematous lungs, in which free expectoration is both difficult and necessary for recovery; and the cases of dropsical effusion, where a mechanical impediment exists either to free circulation or to the expansion of the lungs. It is obvious, from these considerations, that the time during which bloodletting can be beneficially employed in such diseases must be very circumscribed; although it must be admitted, on the other hand, that some cases of all these occur, in which the strength of the circulation is such as to make it safe and beneficial at a much more advanced period than in others. The case of mechanical obstruction to the flow through the heart, from disease of its valves or of the aorta, unconnected with organic alteration either of the lungs or liver, is that in which the repeated loss of blood may generally be best borne.

Much has been said, in some systematic works, of age, sex, temperament, habit of body, habits of life, climate, and season, as influencing the use of this remedy; but the fact is, that there is no age, no sex, temperament, or habit of body; no description of human beings, and no climate or season, in which bloodletting may not, on certain occasions, be performed with advantage,—nay there is none in which its neglect may not be fatal. All that can be said on those heads is chiefly important as pointing out the circumstances in which the indications or contra-indications already stated are chiefly to be expected, but can hardly be said to establish any new rules." 108.

The great class of fevers is then entered upon, Dr. Christison leading the van, and treating of the "general doctrines of fever"—and of "continued fever," when Dr. Shapter breaks the chain, rather abruptly, and interposes "PLAGUE" between continued and intermittent fevers. We think the disease might have fallen in more naturally among the eruptive fevers, than where it is now placed. This, however, is of little consequence. The article on "continued fever" is a very laboured and erudite one, as might have been expected from the pen of Dr. Christison; but there is not—indeed there cannot be, much originality. The criticisms on the opinions of others, however, are candid, and generally correct. We must pass over, with commendation, the papers on "PLAGUE"—on "INTERMITTENT FEVERS," and on "REMITTENT FEVERS," in order that we may take more particular notice of a short essay on "infantile remittent gastric fever," from the practical pen of Dr. Locock. This section makes no pretence to erudite researches among books; and we like it the better on that account, as it makes up at least in originality for the deficiency of book-worming.

This disease has received many names from different authors, according to the theories which they had adopted, or the prominent symptoms which they had observed. The worm fever—mesenteric fever—stomach fever—infantile remittent, &c. are among the chief designations.

"On a careful examination of the history and symptoms, as given by various authors of former and recent times, we are satisfied that much confusion has arisen, sometimes from imperfect attempts to separate into distinct diseases what are in fact but early and later stages of the same, and on the other hand, from an opposite error of confounding what are accidental complications, with what may be considered as the regular and simple form of the complaint." 277.

Dr. Locock thinks that the doctrine of Broussais receives *apparently* "a striking confirmation," in the acknowledged cause of the complaint under consideration. But this confirmation is only *apparent*. Because inflammation of the mucous membrane of the stomach and bowels will cause fever, it does not logically follow that *all fever* depends on this one cause. Inflammation of the cerebral membranes will just as readily produce fever, as that of the gastro-intestinal, and this fact has seemed to afford a striking confirmation of the doctrine of Clutterbuck; yet the profession has some doubts on the subject still. The fact only proves that unity of causation in fever is just as much the "baseless fabric of a vision," as "unity of disease" itself in the brilliant hypothesis of our friend Dr.

Dickson.* This, indeed, is wisely acknowledged by Dr. Locock bimself, who limits the soundness of Broussais's doctrine to this one form of fever—"infantile gastric."

For all practical purposes Dr. L. thinks the division into *acute* and *chronic* will be sufficient. The disease indeed is more uniform than almost any other kind of fever. The description of Dr. Butler applies to the disease as closely now as it did "sixty years since." The following graphic delineation of a single paroxysm we shall quote entire.

1. "*Symptoms of the acute form.* It is not in earliest infancy that this disease is most commonly met with—indeed, many have denied its existence in children during the period of lactation. It is most frequent from the age of two to six, but preserves its peculiar character up to the age of puberty, though, the older the child grows, the less marked are those peculiarities of type. In the *acute* form, the symptoms often come on very suddenly. The child perhaps goes to bed apparently as well as usual, and in an hour is found with a burning skin, a flushed countenance, an injected eye, and a very rapid pulse, varying perhaps from 120 to 160. There is intense thirst, with a dry tongue, which soon becomes coated and covered with a thick white fur; the child is restless and wide awake, often delirious, but able to answer questions or do as directed. If old enough, the child often complains of pain in the head and sometimes in the abdomen, the parietes of which are generally more hot than any other part of the body; indeed, the feet are often cool or cold. There is occasionally sickness and vomiting of sour and offensive, or of greenish or yellow fluid. If the proper remedies be used, in a few hours the skin becomes cool, perspiration breaks out, the tongue is found to be moist, the pulse softer and more quiet, the child falls into a deep and refreshing sleep, and on awaking appears nearly as well as the day before." 278.

Such a speedy and favourable termination, however, can only be expected where the cause was one improper meal, and where the irritating cause is expelled by nature or art. It is rare to find the cause so limited, and the issue so prosperous. Accumulations of improper or of undigested food in the digestive tube will induce a succession of paroxysms, formed only by remissions, instead of intermissions. There is languor and restlessness in the morning, with moist but coated tongue—cool but dry skin—quick pulse—frequently drowsiness, loss of appetite, scanty and high-coloured urine. Towards evening, all the symptoms are exasperated, running the same course as before, and followed by the usual remission. Sometimes there is diarrhœa—sometimes constipation; but in either case the secretions are depraved, and the fœtor resembling that of putrid meat.

"They are dark, pitchy, or clay-coloured, with little or no admixture of bile, or the biliary secretions appear vitiated and unmixed with the general mass. When the bowels have been previously confined, the accumulation of morbid secretions is usually enormous, and dose after dose of active purgatives is necessary to dislodge the offensive load. After the bowels have been cleared out, the dejections are still highly fetid, dark, and slimy, and their character is found to improve with the subsidence of the febrile accessions. In the course of the disease the breath early shows a faint and often an offensive odour, the coat on the tongue becomes more yellow or dirty, and the child is noticed to be frequently

* By the way, Dr. Dickson has placed a very appropriate heading to the title-page of his book—"FALLACIES of Physick."

picking its lips, its nose, the corner of its eyes or its fingers. There is also not uncommonly a short hacking cough. As convalescence approaches, the paroxysms of fever become less marked, take place at a later hour, and last a much shorter time. The intervals of remission are longer, and are indeed almost complete intermissions, the child becoming more lively, returning to its natural habits, and recovering its appetite and strength. In other instances the disease does not come on so suddenly. For a few days the child is heavy and fretful, with disturbed sleep, loss of appetite, and coated tongue; the febrile accessions are very slight and irregular, but go on increasing in length and severity, till the more decided symptoms appear, and run a similar course." 278.

The duration of the acute attack varies from a few hours to a week or a fortnight, after which it gradually subsides into a chronic form, and is then often protracted. The improvement of the secretions is one of the earliest signs of recovery; but for some considerable period a very trifling error indeed will induce relapse:—or neglect of the bowels, too early use of tonics, exposure to damp, or to over-exertion or excitement, will do the same. Violent purgation or drastic purgatives will induce relapses, as well as inattention to the bowels.

The disease is not so entirely void of danger as Dr. Underwood pronounced it. Complication with dysentery or acute inflammation of the bowels will occasionally render the disease fatal.

"The dysenteric complication is indicated by the appearance of the evacuations, which are frequent, attended with violent straining, consist almost entirely of mucus, and are often mixed with blood; while the acute pain in some part of the abdomen, increased on pressure, with retraction of the limbs, early tympanitis, and tendency to constant sickness, indicate the existence of intestinal inflammation. In the acute stage of these complications, the peculiar fetor of the evacuations soon ceases to be constant, and often entirely disappears; but it returns again after a time perhaps, notwithstanding that the inflammatory state of the intestinal mucous membrane has subsided. By the majority of practitioners in this country, however, this absence of fetor of the stools in the progress of the acute form of infantile remittent fever is not considered to be at all uncommon, and they are unwilling to admit the existence of inflammation in such cases. The presence of pain, on the other hand, is by no means a necessary proof of the existence of inflammation, and the tenderness on pressure is often deceptive, as young children, in the fretfulness of disease, are exceedingly impatient of any sort of disturbance, and evince great dislike to pressure on the abdomen or any part of the surface. We must not lose sight also of the fact stated by Andral and others, that even the most severe and fatal forms of intestinal inflammations are often painless." 279.

The etiology of the acute form is traced, directly or indirectly, to "disorder of the digestive organs."

*The Diagnosis* is sometimes false, from confounding the simple fever itself with some of its consequences or concomitants, as worms, gastritis, enteritis, tabes mesenterica, hydrencephalus, &c.

*Treatment.*—When early consulted, Dr. L. prescribes an emetic, especially if there be sickness of stomach. Next morning it will be prudent to administer a smart purgative of calomel, jalap and scammony, or castor oil, senna, or salts, &c., according to the age and constitution of the patient.

"Should the time for an emetic have gone by, from the number of hours which have passed before our assistance is required, we may begin at once with the purgatives, but then it will be generally found necessary to give the calomel alone in the first instance, on account of the irritability of the stomach rendering it difficult for the child to retain medicines which are nauseous or in large quantity, but following it up a few hours after with some other purgative." 282.

Enemata, and the warm bath (if much restlessness obtain) are recommended by Dr. Locock, together with effervescing saline draughts and nitrate of potash. As long as the motions are unhealthy, there will be a recurrence of the fever; but when the secretions begin to assume a natural appearance, we may give the little patient a temporary respite from physick, while meantime the nourishment should be entirely fluid, and unirritating, consisting of farinaceous decoctions, rennet whey, &c.

"It is always necessary to inspect the evacuations, instead of trusting to the report of the nurse. The state of the abdomen on pressure, as to fulness and hardness, must be also attended to, and we should closely watch the indications of pain or tenderness. The fulness and hardness, distinct from collections of air, which are easily detected by percussion, will denote the necessity of following up the use of active purgatives, which may be continued at sufficient intervals, so as not to harass and exhaust the patient, till we are satisfied that the intestines are emptied." 283.

Abdominal tenderness indicates caution in the use of purgatives, unless of the mildest kind, as castor oil or neutral salts—small doses of calomel being combined with hemlock or henbane, and given more frequently.

"The abdomen should now be fomented, and if the pain increases and becomes constant, and the febrile symptoms more permanent, there will be no doubt as to the propriety of applying leeches, which we have found more salutary than general bloodletting. The number of leeches, and the repetition of them, must of course be adapted to the strength and age of the child, as well as to the severity of the symptoms. Many have urged that the disease is so exhausting and so liable to be protracted, that abstraction of blood should be avoided; but it will generally be found that active treatment, pursued judiciously in the early and acute form, will be most likely speedily to arrest the symptoms, and prevent the exhaustion consequent upon the more protracted or chronic form. As the symptoms subside, smaller doses of the remedies recommended may be employed; at more distant intervals, and when the secretions have become healthy, the tongue clean, and the fever subsides, it will be sufficient to give a gentle dose of rhubarb with magnesia or sulphate of potass every other day. At this time the power of the digestion may be assisted by a light vegetable bitter, with ammonia or the other alkalies, twice or three times a day; and we have much confidence in the mineral acids, and especially in Meynsicht's vitriolic elixir (an imperfect ether, formerly much in repute in atrophy and consumption), in doses of from five to thirty drops, according to the age of the child. As the appetite returns, the diet may be slowly and cautiously improved; but it must be always recollected that the slightest excess or carelessness, or any neglect in the management of the bowels, will be likely to be followed by a relapse." 283.

2. *The Chronic form.*—This either succeeds to the acute form, or creeps on slowly and insidiously, from causes similar in kind though slighter in degree, than those which lead to the acute fever. Long habits of indulging in improper food—swallowing it too fast—inattention to the bowels—exposure to damp—and insufficient exercise, are the most prominent causes of chronic gastric fever. Butter believed it to be epidemic and contagious; but the latter supposition is now clearly exploded. It often succeeds to certain infantile diseases, as hooping-cough, measles, scarlatina, &c. &c.—probably in consequence of the gastro-intestinal derangement produced by them. The symptoms resemble those of the acute disease, except that they are milder in degree, and less marked in their character. Although the paroxysms are less intense, they are longer in duration, and the intervals less free from irritation. The fæcal accumulations are less in bulk, and the fætor less offensive; but still the secretions are rarely natural—the abdomen is hot, and sometimes tender to the touch, as well as tumid from flatulence—the tongue furred in the middle, but red and angry at the sides—teeth covered with sordes—lips parched—skin harsh and dry, and ultimately wrinkled from emaciation—urine scanty and high-coloured, with white sediment—breath offensive—frequently a hacking cough, with picking of the nose, and indeed of every part of the body.

" In the advanced stages the fretfulness of the child is often most distressing, or sometimes it lies for hours taking little or no notice of any thing, and either apparently dozing with half-closed eyes, or when roused immediately resuming the incessant picking. At this period the appetite is very often craving, and the child evinces great irritation and distress on being denied food. In other cases there is urgent thirst, but the appetite is lost; in others, again, there is neither appetite nor thirst, so that there is great difficulty in getting the child to swallow any thing, from its dislike to be disturbed. In the still more severe cases there is generally some complication, either diarrhœa or dysentery, when the mucous lining of some portion of the intestines is found either softened, abraded, or the intestinal follicles enlarged and in various stages of ulceration. It is still more common to find that mesenteric disease is excited by the extension of the irritation to those glands; when this happens the abdomen is hard and tumid, and the enlarged glands may often be felt through the parietes of the abdomen. This complication is more apt to occur where there is a scrofulous taint, and in such constitutions tubercular disease in the lungs occasionally supervenes. Instances of death without one or other of these complications are rare, and even when the child appears to be reduced to the lowest degree of emaciation and debility, by proper treatment recovery may be effected." 284.

*Treatment of the Chronic form.*—Most of the remedies for the acute form are necessary in the chronic; but they must be administered with much more caution, and in much smaller doses. Active treatment in chronic diseases is productive of much mischief in the sick room. In this form of the complaint drastic purgatives are too often employed, and the consequence not unfrequently is, that irritation is converted into inflammation, or the chronic form into the acute—with dysenteric symptoms and bloody stools.

In other cases, softening of the mucous membrane takes place, and ulceration follows.

" If, in the first instance, we have reason to suspect accumulation in the intestines, an active purgative, containing calomel, may be safely and advantageously administered; nor is there any objection to have such more powerful medicine occasionally repeated, especially if, by any error of diet or negligence in the management, a relapse or an increase of the symptoms have taken place.

But after the purgatives have sufficiently cleared the bowels, the secretions will be best improved by milder remedies: mercurials in gentle doses, especially the Hydrarg. cum Cretâ, or calomel rubbed down with chalk, should be given every night, or every other night. A combination of the mercurial with the diaphoretic, particularly ipecacuanha, or, if much restlessness, with Dover's powder, will be found very useful. A mild purgative on the following morning, such as castor oil, compound decoction of aloes, rhubarb and magnesia, or rhubarb with sulphate of potass will be necessary. Denman, Underwood, Butter, and Pemberton, have all spoken highly of this latter preparation, considering it peculiarly adapted to meet the indications by relieving the fever, improving the secretions, and quickening the action of the bowels and kidneys. The quantities must of course be adapted to the age and strength of the patient, but from two to three evacuations will be desirable daily; a larger number will exhaust the child, and fewer will scarcely keep the bowels sufficiently free from offending matters. Saline medicines at intervals will also be beneficial; and the addition of henbane, hemlock, or lettuce, has been found by most practitioners to allay the general irritation, compose the restless distress of the child, and render the action of the remedies more genial. Frictions with oil over the abdomen, or with slightly stimulating lotions, will often be of use, and we have especially observed advantage from the nitro-muriatic acid largely diluted. A nightly warm bath should not be omitted; it promotes perspiration, and relieves the mucous surface, besides composing the child, and contributing to its comfort.

The diet during the early treatment should be such as will most easily assimilate, and will be least likely to produce irritation, if only partially digested; for this purpose we may allow chiefly barley-water, rennet whey, thin arrow-root, or other farinaceous gruels, and weak chicken or veal broth. When there is thirst, soda-water or toast and water, or slightly acidulated beverages, may be taken." 285.

When all febrile symptoms have subsided, the tongue has become clean, and the secretions healthy—and not till then, should the mildest tonics be used. Dr. Locock recommends the mineral acids, as infusion of roses, to which some of the neutral aperient salts may sometimes be added. We are partial to the bitter infusions with soda. It is only after decided convalescence has set in that the more powerful tonics can be safely employed. Great care is necessary in respect to diet for weeks or even months after recovery, since relapses are very apt to recur from the most trifling irregularities. We need hardly add, in taking leave of this article, how much it speaks for the practical judgment and experience of its author.

The subject of "HECTIC FEVER" is briefly but judiciously treated by Dr. Christison—"Small Pox" is ably handled by Dr. Gregory—and Measles and Scarlatina have been allotted to Dr. George Burrows, who has discharged his duty well. In "PUERPERAL FEVER," Dr. Locock again comes on the stage, and, in the brief space of nineteen pages, has discussed a "quæstio vexata" that has occupied many ponderous volumes, and called forth the acerbity and odium medicum of many eminent physicians and surgeons.

"On perusing the numerous treatises that have been published within the last half century on this highly important class of diseases, the reader must necessarily be struck with the very extraordinary difference of opinion amongst the several writers as to the history and nature of the disease, the symptoms, mode of treatment, and the result of the practice adopted. The only point on which all seem to be agreed is, its great and striking fatality, and that it is one of the most serious, intractable, and destructive maladies to which puerperal women are liable." 348.

Dr. L. thinks that all these discrepancies may be easily reconciled, without attributing "mala fides" to any author. Each has described what he has seen—but they all saw different forms or shades of the same disease—and hence their clashing delineations. From Dr. L.'s observations and researches, he is not inclined to subscribe to the doctrine that connects puerperal fever with local inflammation. But even if this doctrine were admitted, we should never be able to trace the inflammation to any one locality in all cases—which is, indeed, the case with all other fevers.

"In considering puerperal fevers it has long been our conviction, that what has been called by Sydenham *the constitution of the year*, has been too much lost sight of. The great difference in the accounts of puerperal fevers by different writers is thus easily explained." 349.

Dr. L. treats the disease under five heads—1. Acute Puerperal Peritonitis.—2. Adynamic, or Malignant Puerperal Fever.—3. Puerperal Intestinal Irritation.—4. False Peritonitis.—5. Milk Fever. Our closing limits prevent us from more than glancing at the third head. Puerperal intestinal irritation is by no means a rare form of the disease under consideration. It is of great consequence to distinguish it, for a mistake in the diagnosis may be of serious consequences. It is not, in itself, inflammatory; nor does it *necessarily* lead to inflammation. To treat it for inflammation, which is too often done, may risk the life of the patient. It is a frequent *cause* too, of other forms of puerperal fever, and not seldom *accompanies* them.

"At any period after delivery, where the bowels have been previously neglected or mismanaged, this affection may come on. The symptoms are more gradual in their progress at first; there is general uneasiness, scarcely describable by the patient, often for some days before the more marked symptoms make their appearance; the appetite fails; the tongue becomes coated with either a creamy, or a dirty white; the skin is over cool for part of the twenty-four hours, and that state alternates with irregular febrile heat, accompanied often with headach, and generally a quick pulse; there is frequently deep-seated uneasiness in the abdomen, which is full and rather tense, often rather tender to pressure, but not generally to firm steady pressure; there is a frequent feeling of sickness, and often vomiting; sometimes this vomiting is profuse and incessant, and the fluid ejected is dark and offensive; in many instances this is thrown off the stomach with little or no effort, but apparently from the effect of flatulence. A very common symptom, more especially after the first day or two, is diarrhœa; the evacuations are dark, fetid, watery or slimy, with much flatulence, fetor of the breath, and increased abdominal tenderness; the pulse increases in rapidity; the exacerbations of fever are of longer duration, and attended with great prostration of strength and feeling of despondency; the tongue indicates subacute gastric inflammation—it is sometimes white in the centre with florid edges and tip, the bright red, angry-looking portion suddenly emerging from the border of the white coat; at other times the white or yellow coat entirely disappears, and the whole tongue is left morbidly red, shining, and glossy; in some cases perfectly glazed; in others it is rough, and as it were scalded, the mucous membrane of the mouth being at the same time often covered with aphthæ. The strength of the patient rapidly diminishes under the exhausting diarrhœa and the continual or irregular fever, and death is generally preceded by some of the symptoms of the other forms of puerperal fever. Many of these cases are treated by bleeding, on the supposition that they are inflammatory, but bleeding only aggravates the symptoms. They are also more likely to arise after labours which have been unusually protracted, or where uterine hæmorrhage has occurred to a great extent. In the latter instances, besides the symptoms described, there is much affection of the head; acute pain, with strong pulsation in the centre, confused noises, want of sleep, low delirium, and constant restlessness. In spite of the palpable cause of these distressing sensations, it is by no means uncommon to find this form of child-bed fever mistaken for vascular plethora, and the temporary relief to the head, obtained by the local abstraction of blood, has often led to a repetition of exhausting remedies." 362.

In several fatal cases Dr. L. observed the entire absence of all organic changes. There was much flatus in the intestines and stomach—all the tissues being pale and bloodless, with venous congestion in the brain. In some of the more protracted cases, attended with diarrhœa, there was ulceration of the mucous membrane of the bowels.

" It is most important to detect this form of mischief early, and to distinguish it from the more formidable conditions of the puerperal state. They are, however, so often blended or complicated, as to increase materially the difficulty. When uncomplicated, the chief points to be noticed are, the insidious character and slow progress of the symptoms, the state of the tongue, and the condition of the secretions. The absence of acute pain and tenderness is not much to be depended upon, as it has been already observed that those symptoms are not always present in the peritoneal fevers. The irritable state of the tongue, the peculiarly foul and offensive evacuations, and the subsequent diarrhœa, when existing along with the signs of inflammatory mischief in the abdominal or pelvic cavity, whether of the sthenic or malignant character, are all to be taken as evidences of the complication of this state of intestinal irritation with the genuine puerperal fever." 363.

Dr. L. limits his observations on the treatment to the simple form of *irritation*, uncomplicated with inflammation. The first indication is obviously to clear the bowels of offending matters—to improve the secretions—and to sustain power without increasing fever.

"When we are called to such a case early, a full dose of calomel, James's powder, and opium, may be given, followed in four or five hours by castor oil, which is generally the most efficient and the least irritating purgative. If there be much sickness, so that such remedies will not be retained, from five to ten grains of calomel alone will almost always allay the vomiting; after which a large enema of gruel and castor oil may be injected. Several successive doses of purgatives will generally be required to remove the scybala and offensive accumulations from the intestines, and a change of purgative will frequently accomplish this, when the first has latterly brought away nothing but watery motions. The repetition of the purgatives must depend on the strength of the patient and on the effect produced, the full state of the abdomen, when felt by the hand, being our guide as to the existence still of an unremoved load. When diarrhœa is an early symptom, or at least has begun before we see the patient, we shall do little good in our efforts to restrain it, unless we give these active purgatives. Chalk mixtures and astringents only aggravate the symptoms in the first instance, the cause which keeps up irritation still remaining. After we have succeeded in cleansing the bowels, milder alteratives will be sufficient. The hydrargyrus cum creta, or small doses of calomel combined with ipecacuanha and prepared chalk, may be given at short intervals, interposing some mild laxative, as rhubarb and magnesia, or castor oil, once in one or two days. Where the patient has been much exhausted, an enema of gruel may be given; and if there is much soreness of the lining membrane, laudanum or tincture of henbane may be added. Opium may be combined with the alteratives, especially where the diarrhœa still continues profuse. We have often found advantage in this state, from occasional very small doses (eight to ten grains) of sulphate of magnesia in some spiced water, combined with five or six drops of laudanum. Sometimes the nitric or sulphuric acid with laudanum effectually restrains the diarrhœa, and improves the character of the tongue, especially if there are aphthous ulcerations. The usual astringent remedies are inadmissible in the commencement, and only to be employed if the diarrhœa persist after the bowels have been cleared. The diet should be nourishing, but not stimulating, unless there is very great exhaustion. Arrow-root, gruel, broth, jelly, and other bland articles are the safest. Milk with soda or Seltzer water is very grateful to the patient in allaying thirst and keeping up power, especially where there is sickness. As the diarrhœa subsides, and the secretions become healthy, more nourishment may be taken; but any hurry in this respect, and any carelessness in the nature of the diet, often lead to relapses. Infusions of cascarilla or cinchona, with either ammonia or the mineral acids, may be tried in the convalescent stage, and pure air will be then of great advantage. In cases where the head affection follows exhausting labours, with or without other signs of intestinal irritation, still greater caution is necessary in the treatment. In both, from a disposition to be too acutely alive to all ailments after parturition, there is often an eagerness to use active remedies, and especially to bleed." 364.

The volume is closed by a long article on diseases of the skin, occupying seventy pages. We should have thought that England might have furnished a labourer for this vine-yard, since many of our physicians and surgeons have distinguished themselves in dermological researches. The editor, however, has laid M. Schedel, of Paris, under contribution for the article in question; and we are bound to acknowledge that it is constructed with equal care and ability. M. Schedel, indeed, has attained reputation in this branch of medical science long before his appearance on the English stage. If the work be conducted with the talent and research displayed in this first volume, it will command success.

